# Clozapine N-oxide, compound 21, and JHU37160 do not influence effortful reward-seeking behavior in mice

**DOI:** 10.1007/s00213-023-06465-w

**Published:** 2023-10-04

**Authors:** Yoshiatsu Aomine, Yoshinobu Oyama, Koki Sakurai, Tom Macpherson, Takaaki Ozawa, Takatoshi Hikida

**Affiliations:** 1 Laboratory for Advanced Brain Functions, Institute for Protein Research, Osaka University, Suita, Osaka, Japan; 2 Department of Biological Sciences, Graduate School of Science, Osaka University, Toyonaka, Osaka, Japan; 3 Laboratory of Protein Profiling and Functional Proteomics, Institute for Protein Research, Osaka University, Suita, Osaka, Japan

**Keywords:** DREADDs, CNO, Compound 21, JHU37160, Clozapine, Operant licking, Progressive ratio schedule, Break point, Food seeking, Motivation

## Abstract

**Rationale:**

Clozapine N-oxide (CNO) has been developed as a ligand to selectively activate designer receptors exclusively activated by designer drugs (DREADDs). However, previous studies have revealed that peripherally injected CNO is reverse-metabolized into clozapine, which, in addition to activating DREADDs, acts as an antagonist at various neurotransmitter receptors, suggesting potential off-target effects of CNO on animal physiology and behaviors. Recently, second-generation DREADD agonists compound 21 (C21) and JHU37160 (J60) have been developed, but their off-target effects are not fully understood.

**Objectives:**

The present studies assessed the effect of novel DREADD ligands on reward-seeking behavior.

**Methods:**

We first tested the possible effect of acute i.p. injection of low-to-moderate (0.1, 0.3, 1, 3 mg/kg) of CNO, C21, and J60 on motivated reward-seeking behavior in wild-type mice. We then examined whether a high dose (10 mg/kg) of these drugs might be able to alter responding.

**Results:**

Low-to-moderate doses of all drugs and a high dose of CNO or C21 did not alter operant lick responding for a reward under a progressive ratio schedule of reinforcement, in which the number of operant lick responses to obtain a reward increases after each reward collection. However, high-dose J60 resulted in a total lack of responding that was later observed in an open field arena to be due to a sedative effect.

**Conclusions:**

This study provides definitive evidence that commonly used doses of CNO, C21, and J60 have negligible off-target effects on motivated reward-seeking but urges caution when using high doses of J60 due to sedative effects.

**Supplementary Information:**

The online version contains supplementary material available at 10.1007/s00213-023-06465-w.

## Introduction

Designer receptors exclusively activated by designer drugs (DREADDs) are a chemogenetic tool that allows control of the activity of targeted neuronal populations via a combination of conditionally expressed designer receptors and targeted designer drug administration in free-moving laboratory animals (Armbruster et al. 2007; Roth 2016). As such, they are widely used to investigate the function of specific neuronal populations in various animal behaviors. Commonly used DREADDs, such as hM3Dq (stimulatory) and hM4Di (inhibitory), were developed by modifying human muscarinic receptors so that they specifically respond to the synthetic ligand clozapine N-oxide (CNO) (Armbruster et al. 2007), but not to their endogenous ligands. CNO, a metabolite derivative of the well-known atypical antipsychotic clozapine, was initially selected as the exogenous ligand for both these receptors as it was considered to be pharmacologically inert, lacking affinity for various endogenous receptors (Weiner et al. 2004). However, several problems regarding the specificity of CNO to DREADD systems have been identified in recent years.

Although the lack of off-target effects of CNO at <1-μM concentrations has been reported (Armbruster et al. 2007), 10-μM CNO has been suggested to potentially bind to various endogenous receptors, including monoaminergic (dopamine, serotonin, histamine) and muscarinic receptors (Gomez et al. 2017; Appl et al. 2012; Jendryka et al. 2019). Additionally, studies in primates, rats, and mice have revealed that CNO does not penetrate the blood-brain barrier (BBB) as effectively as initially appreciated. To permeate the BBB, CNO is actually reverse-metabolized to clozapine, an antipsychotic with various psychotropic effects, and then crosses the BBB and functions as the de facto DREADD ligand in the brain (Gomez et al. 2017; Lin et al. 1996; Manvich et al. 2018; Martinez et al. 2019; Raper et al. 2017). In rats and mice, around 7.5 to 13% of administered CNO has been found to be reverse-metabolized to clozapine (MacLaren et al. 2016; Manvich et al. 2018). Most likely due to these unexpected mechanisms, the administration of CNO is known to alter animal neurophysiology and behavior, resulting in decreased striatal glutamate levels, decreased anxiety-like behavior, reduced acoustic startle reflex, and attenuated d-amphetamine-induced hyperlocomotion (Bærentzen et al. 2019; MacLaren et al. 2016; Tran et al. 2020).

Recently, a new generation of DREADD ligands has been developed, including compound 21 (C21) and JHU37160 (J60), JHU37152, and deschloroclozapine (Chen et al. 2015; Bonaventura et al. 2019; Nagai et al. 2020). In particular, C21 and J60 have seen increased use in rodents in recent years. C21 was developed as a highly hM3Dq-selective ligand (when compared with CNO), is not reverse-metabolized, passes the BBB at adequate levels, and has lower binding affinity for endogenous receptors than clozapine (Chen et al. 2015; Thompson et al. 2018). However, a previous study has suggested that C21 may act as an antagonist for endogenous receptors, as it shows over 80% competitive inhibition of endogenous ligand binding to monoaminergic (dopamine, serotonin, histamine), muscarinic, and adrenergic receptors (Jendryka et al. 2019). J60 was designed as a ligand that exhibits even higher DREADD affinity and brain occupancy than C21 in both rodents and monkeys. However, J60 shows similar binding profiles to clozapine at dopamine and muscarinic receptors (Bonaventura et al. 2019). For this reason, Bonaventura et al. (2019) have suggested that, similar to clozapine, the dose of J60 should be adjusted to minimize off-target effects.

As we described above, various off-target effects of DREADD ligands on monoaminergic systems and animal behaviors have been reported. However, their possible effects on monoamine-dependent appetitive behaviors have rarely been tested. Here, we focused on motivated reward-seeking behavior, which is highly dependent on monoaminergic neurotransmitter systems in the brain (Bromberg-Martin et al. 2010; Fischer and Ullsperger 2017; Torrealba et al. 2012), to test the possible off-target effects of various DREADD ligands. Motivated behavior is commonly quantified in laboratory animals using effortful reward-seeking tasks such as a progressive ratio (PR) schedule of reinforcement, where the animals have to perform effortful and sustained operant behaviors to obtain rewards. Until now, it has been shown that systemic administration of a moderate dose of CNO (0.3 to 1 mg/kg) in rats had no effect on motivated reward-seeking behavior under a PR schedule (Minnaard et al. 2022). In the present study, we systematically tested the effects of CNO, C21, and J60 DREADD ligands administered at a wide range of doses (0.1 to 10 mg/kg) on motivated reward-seeking behavior in mice, by assessing sustained instrumental responses to obtain liquid rewards under a PR schedule in a self-paced operant licking task.

## Materials and methods

### Animals

Eight-week-old female (*n*=12) and male (*n*=12) C57BL6/JJcl mice (CLEA Japan Inc., Tokyo, Japan) were used for all experiments. Mice were housed on a 12-h light/dark cycle (light: 0800–2000, dark: 2000–0800) in a quiet environment with room temperature maintained at 24 °C ± 2 °C and 50 ± 5% humidity. Mice were housed according to sex, with ad libitum access to food and water until the start of behavioral experiments. All animal experiments complied with institutional guidelines set by Osaka University Institute for Protein Research Animal Committee.

### Drugs

Clozapine N-oxide (CNO) dihydrochloride (water soluble) (HB6149, Hello Bio, Bristol, UK), DREADD agonist 21 (compound 21 (C21)) dihydrochloride (water soluble) (HB6124, Hello Bio, Bristol, UK), and JHU37160 dihydrochloride (DREADD ligand) (water soluble) (HB6261, Hello Bio, Bristol, UK) were dissolved in saline. All drug concentrations were designed and prepared to be delivered at 10 ml/kg via intraperitoneal (i.p.) injection.

### Pre-training (fixed ratio (FR) schedule operant task)

First, mice were food-restricted to maintain them at 80–90% of their initial free-feeding body weight. During the entirety of the experiment, mice were fed daily after the completion of testing, or at a similar time (1500–2000) on non-test days. After 7 days of food restriction, mice began pre-training under an FR schedule in behavioral operant chambers (Modular Test Chamber ENV-307W-CT, Grid Floor ENV-307W-GFW, Contact Lickometer Controller ENV-250, Med Associates Inc., USA). Mice started training under an FR-3 schedule, in which three operant licks led to the delivery of a liquid reward (10% sucrose solution, 5 μl) via a drinking spout (KN-348-18G-50, Natsume Seisakusho, Japan) that was connected to a liquid receptacle by a silicone tube attached to an electronic pinch valve to control delivery (HYN-3-DC24V, CKD, Japan). Training was conducted once daily in a 30-min session until mice could obtain 30 rewards in a session, at which point they progressed to an FR-10 schedule for the next session in which 10 operant licks were required to obtain each reward. As with the FR-3, mice finished FR-10 pre-training once they could obtain 30 rewards in a 30-min test session. All mice were able to complete FR-3 and FR-10 pre-training within a single session at each schedule, respectively.

### Drug administration in progressive ratio (PR) schedule operant tasks

**Fig. 1:**
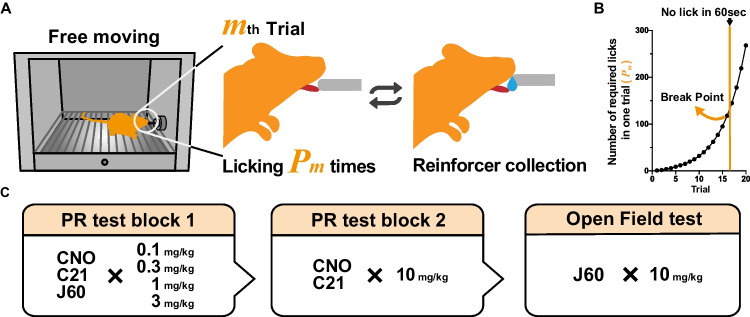
**A** Illustration of progressive ratio (PR) operant licking task. *m* is the order number of a trial and *P*_*m*_ is the response ratio. **B** The response ratio schedule of the PR schedule test. **C** The order of experimental test blocks

The PR tasks were conducted as previously described (Aomine et al. 2022), with some modifications of operant chamber to measure licking. In the PR schedule, the number of licks required to obtain rewards (response ratio, *P*_*m*_) was determined according to the following formula (e.g., the reward was given after a single lick in the first trial, but the number of licks required for consecutive reward presentation increases gradually; i.e. 1, 2, 4, 6, 9, 12, 15, 20, …) (Richardson and Roberts 1996) where *m* is the trial number (Fig. 1A and B).

$${P}_m=\left[5{e}^{\left(m\times 0.2\right)}\right]-5$$

To assess motivation to obtain a reward, we calculated the virtual endpoint of the session, which is the length of time until the mouse failed to lick the spout for over 1 min, or a maximum time of 30 min. The response ratio (*P*_*m*_) for the last reward collection was designated as the break point (limit of the effort the animal will expend to gain the reward).

Following completion of FR-10 pre-training, mice were trained under a PR schedule for 2 days, in which they were administered i.p. saline 30 min prior to each session, in order to habituate animals to the PR schedule and i.p. injections. After habituation, three drug administration tests were conducted. In test block 1, the effects of low-to-moderate doses (0.1, 0.3, 1, and 3 mg/kg) of DREADD agonists (CNO, C21, J60), as well as administration of saline as a control group, were tested. In test block 2, the effects of a high dose (10 mg/kg) of CNO, C21, or J60, as well as administration of saline as a control group, were tested. The order of drugs or saline control sessions tested within each of the test blocks was counterbalanced. All drugs were injected i.p. 30 min before the experiment. Each test day was separated by 2–3-day intervals in order to minimize lingering effects of the drugs from the previous test session (Traut et al. 2023).

### Drug administration in the open field test

Food restrictions were removed when all PR schedule tests were completed. Starting 5 days after the removal of food restrictions, open field tests were initiated. In this test, the spontaneous locomotion of mice was measured in an open field test chamber (infrared beam type, Med Associates Inc., USA) via infrared beams located on the *X*, *Y*, and *Z* axes. Chambers were illuminated with LED light (300 lux), and distance moved in meters was measured for 60 min. Mice were placed in the test chamber immediately after i.p. administration of J60 10 mg/kg or saline in a counterbalanced schedule at 5-day intervals.

### Statistical analysis

GraphPad Prism software (v. 8.0.1, GraphPad Software Inc.) was used for all statistical analyses. Repeated measures ANOVAs were used for break point and total lick analyses and post hoc Holm-Šídák’s multiple comparison tests for comparisons with saline or vehicle. The Gehan-Breslow-Wilcoxon tests with the Bonferroni multiple corrections were used for survival curve analyses. Two-way repeated measures ANOVAs with post hoc Holm-Šídák’s multiple comparisons tests were used for analysis of locomotion in the open field test.

## Results

### Low-to-moderate doses of CNO, C21, and J60 do not affect reward-seeking behavior (PR test block 1)

**Fig. 2:**
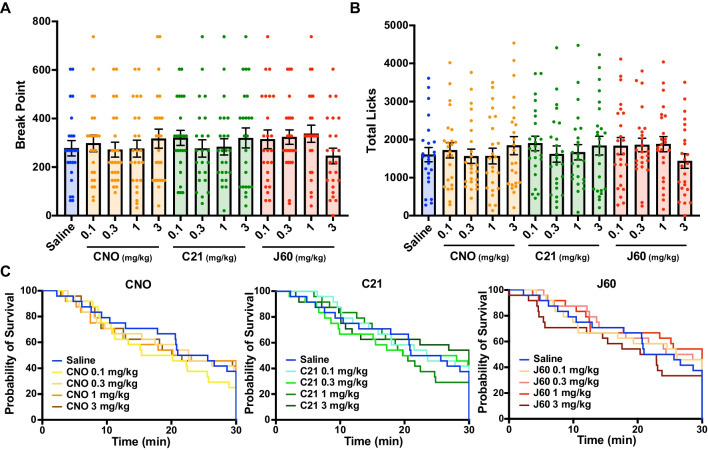
CNO, C21, and J60 at low-to-moderate doses (0.1–3 mg/kg) had no effect on performance of the PR schedule test. **A** Break point: number of licks required to obtain the last reward at the time when the mouse finishes the session. **B** The total number of licks in the session. **C** Survival curve of the duration until the end of the session (no lick for 60 s, or end of the 30-min session). Data represent the mean ± SEM

Following PR schedule habituation, mice were again tested under a PR schedule after administration of various doses of CNO (0.1–3 mg/kg), C21 (0.1–3 mg/kg), J60 (0.1–3 mg/kg), or saline. These doses were chosen as they represent low-to-moderate dose ranges commonly used in DREADD experiments (Goutaudier et al. 2019; Roth 2016). No significant effects of the drugs on break point or total licks during the 30-min test were found by a repeated measures ANOVA (Fig. 2A and B, break point: *F* (7.767, 178.6) = 0.7081, *p* = 0.68, total licks: *F* (7.699, 177.1) = 0.6226, *p* = 0.752). In post hoc multiple comparison analysis, there were no significant differences between each drug and its concentration compared to saline (Supplementary Table 1). Analysis of the survival curves of the sessions revealed no significant effect of drug concentrations (Supplementary Table 1, all adjusted *p*-values > 0.9999) on session length. These results indicated that administration of CNO (0.1–3 mg/kg), C21 (0.1–3 mg/kg), and J60 (0.1–3 mg/kg) at low-to-moderate doses did not affect motivated reward-seeking behavior under a PR schedule.

### High-dose CNO and C21 do not affect reward-seeking behavior (PR test block 2)

**Fig. 3:**
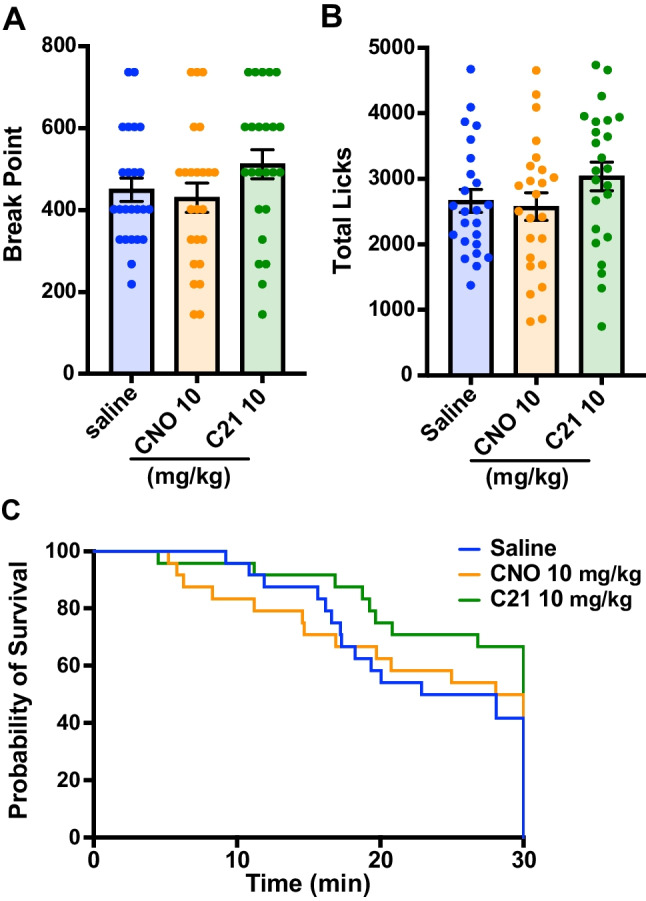
CNO and C21 at a high dose (10 mg/kg) do not have effect on performance in the PR schedule test. **A** Break point: number of licks required to obtain the last reward at the time when the mouse finishes the session. **B** The total number of licks in the session. **C** Survival curve of the duration until the end of the session (no lick for 60 s, or end of the 30-min session). Data represent the mean ± SEM

As no effect of low-to-moderate doses of DREADD ligands was observed in test block 1, it was next examined whether a high dose (10 mg/kg) of CNO, C21, or J60 might alter responding under a PR schedule. While no significant effect of high-dose CNO or C21 was found on break point or total licks in the PR schedule test by a repeated measures ANOVA (Fig. 3A and B, break point: *F* (1.928, 44.34) = 2.384, *p* = 0.1057, total licks: *F* (1.936, 44.54) = 2.033, *p* = 0.1442), high-dose J60 was found to result in a complete lack of responses (data not shown), and mice were observed to be motionless in the operant chambers. Post hoc multiple comparison analysis revealed no significant difference between saline and CNO or C21 on break point and total licks (break point: saline vs CNO 10 mg/kg *p* = 0.7186, saline vs C21 10 mg/kg *p* = 0.2453; total licks: saline vs CNO 10 mg/kg *p* = 0.6127, saline vs C21 10 mg/kg *p* = 0.2256). Similarly, there was no significant effect of CNO or C21 on the survival curves of the sessions (Fig. 3C, saline vs CNO 10 mg/kg: *p* > 0.9999, saline vs C21 10 mg/kg: *p* = 0.1822). These results indicated that even at high doses (10 mg/kg), CNO administration does not alter motivated reward-seeking behavior in mice. However, our findings indicate that high doses of J60 are not appropriate for use in operant tasks as they abolish responding.

### High-dose J60 reduces spontaneous locomotion

To investigate whether the lack of responses in the PR schedule test that was observed after high-dose J60 administration was due to a sedative effect, we next measured the effects of high-dose J60 on spontaneous locomotor activity. Mice were tested in an open field arena immediately after being administered a high dose (10 mg/kg) of J60 (Supplementary Figure 1A). A two-way repeated measures ANOVA revealed significant main effects of drug (J60) and time, along with a significant interaction between these factors (Supplementary Figure 1B, drug: *F* (1, 7) = 26.87, *p* < 0.0001, time: *F* (11, 77) = 28.24, *p* = 0.0013, drug × time: *F* (11, 77) = 5.061, *p* < 0.0001). Post hoc analysis showed a significant reduction of spontaneous locomotor activity in the J60-administered condition compared to the saline control condition after 10 min from the start of the test, and J60 treatment completely reduced mouse locomotion by 25 min after administration (saline vs J60 10 mg/kg, 0–5 min: *p* = 0.2196, all remaining time bins p < 0.0001). These results indicate that i.p. administration of J60 at a dose of 10 mg/kg completely inhibit spontaneous locomotor activity in mice, indicating that J60 at high doses is sedative and inappropriate for use in behavioral tasks.

## Discussion

Here, for the first time, we systematically examined the off-target effects of DREADD ligands CNO, C21, and J60 on motivated reward-seeking behavior in mice. Operant responding under a PR schedule was measured for 30 min after i.p. administration of CNO, C21, or J60 at a wide range of doses (0.1, 0.3. 1, 3, 10 mg/kg). At low-to-moderate concentrations (0.1–3 mg/kg), neither CNO, C21, nor J60 had significant effects on motivated reward-seeking behavior. Similarly, high concentrations (10 mg/kg) of CNO and C21 had no significant influence on motivational reward-seeking behavior, but high-dose J60 was able to completely abolish responding. These results suggest that even at a dose of 10 mg/kg, CNO and C21 are appropriate for use as a DREADD ligand in tests of motivated reward-seeking without undesirable off-target effects. Furthermore, our studies demonstrate that while low-to-moderate doses (0.1–3 mg/kg) of J60 are also appropriate for use in such behavioral tests, high doses (in the 10 mg/kg range) should not be used for behavioral experiments in mice as they result in sedation.

Since the development of DREADDs, CNO has been the most commonly used DREADD ligand. However, it has recently been shown that following CNO administration, reverse-metabolized clozapine is actually the main compound activating DREADDs, and the amount of clozapine present in the brain is much higher than that of CNO (Gomez et al. 2017; Raper et al. 2017). This is potentially a problem as clozapine has been reported to alter a wide range of behavioral tests, including measures of locomotion and anxiety, in mice at doses as low as 0.05–0.1 mg/kg (Ilg et al. 2018). However, our study found no off-target effects of CNO at any of the tested doses (0.1 to 10 mg/kg) on break point or total licks in a PR schedule test in our study. These findings support those of another recent study reporting that CNO doses from 0.3 to 1 mg/kg are safe to use for behavioral tests of motivated reward-seeking in mice (Minnaard et al. 2022) and indicate that this appropriate dose range can be extended to include doses of up to 10 mg/kg.

C21 is not reverse-metabolized to clozapine, can pass the BBB by itself, and has a high hM3Dq selectivity compared to CNO. It has been known that moderate doses of C21 (1 to 3 mg/kg) are sufficient to activate virally expressed DREADDs in the brain to change behaviors (Kang et al. 2020; Lafferty et al. 2020). However, recent pharmacokinetic profiling has shown significant competitive binding of C21 to monoamine (dopamine, serotonin, histamine), opioid, muscarinic, and adrenergic receptors (Chen et al. 2015; Jendryka et al. 2019; Thompson et al. 2018). Most likely due to such widespread binding properties, C21 (0.5 mg/kg) has been reported to increase spontaneous spiking activity of rat SNc neurons in vivo, although previous findings have not shown any visible effects on behavior (Goutaudier et al. 2020; Kang et al. 2020; Lafferty et al. 2020; Tran et al. 2020). We administered 10-mg/kg doses of C21, the maximum dosage seen in previous studies (Thompson et al. 2018), and still found no alteration in motivated reward-seeking behavior. This result suggests that even at doses much higher than those necessary to activate DREADDs, C21 can be used to evaluate motivated reward-seeking behavior.

J60 is a novel DREADD ligand with high in vitro potency compared to clozapine and C21. After administration, J60 is known to effectively permeate into the brain from blood circulation, as evidenced by eightfold higher concentrations found in the brain than in serum (Bonaventura et al. 2019). This is in contrast to C21, which exists in serum at higher concentrations compared to the brain after administration. This characteristic of J60 reduces the required dose for use as a DREADD ligand, usually to around 0.1 mg/kg. Previously, no off-target effects have been reported at 0.1 mg/kg (Barbano et al. 2020; Bonaventura et al. 2019; Costa et al. 2021; Zhang et al. 2020). We extended these previous findings by confirming that at low doses (0.1 to 3 mg/kg), J60 does not affect motivated reward-seeking behavior. However, in our study, a high dose (10 mg/kg) strongly inhibited spontaneous locomotor activity in mice. To our knowledge, this is the first report of an off-target effect of J60 on behavior.

While the mechanism by which high-dose J60 causes sedation is still unclear, it is known that J60 has a very similar binding profile to clozapine, which hinders spontaneous locomotor activity at doses greater than 5 mg/kg via binding at 5-HT2A receptors in the forebrain (Bonaventura et al. 2019; McOmish et al. 2012). Thus, it is possible that binding at 5-HT2A receptors in the forebrain may also underlie the suppression of locomotor activity observed at a high dose (10 mg/kg) of J60.

The aforementioned differences in the sedative properties of DREADD ligands most likely result from variations in their pharmacokinetic (BBB permeability) and pharmacodynamic (binding affinities to each receptor) properties, both complex characteristics influenced by various chemical features of the compound. DREADD ligands described in this paper have a fundamental structure consisting of a benzodiazepine tricyclic core and a piperazine ring (Supplementary Figure 2). Chen et al. (2015) have shown that modifications at the para position of the piperazine ring are important in determining potency against DREADDs. In particular, CNO is known to exhibit very poor BBB permeability due to the presence of the N-oxide group on the piperazine ring, causing it to be expelled from the cell by P-glycoprotein (P-gp) efflux pumps that remove foreign hydrophobic molecules from the cell (Gomez et al. 2017). Both J60 and clozapine exhibit low serum-brain ratio (i.e. high BBB permeability) (Bonaventura et al. 2019) and have a hydrophobic modification to the piperazine ring (J60, ethyl group; clozapine, methyl group). C21 has moderate permeability and lacks any modifications to the piperazine ring (Bonaventura et al. 2019; Jendryka et al. 2019). Such knowledge of BBB permeability from past studies seems to be well in line with the severity of sedative effects observed in this study, where highly BBB permeating J60 resulted in sedation, whereas CNO and C21 with low-to-medium permeability did not have any effects on locomotion, even at high doses. Consideration of BBB permeability is necessary when determining appropriate doses for each ligand and calls for further studies which exhaustively examine the relationships between chemical structure, BBB permeability, and behavioral effects.

In summary, we systematically investigated the effects of three different DREADD ligands, CNO, C21, and J60, on motivated reward-seeking behavior under a PR schedule of reinforcement in a operant licking task. All three DREADD ligands were found to have no off-target effects on performance of the task at concentrations widely used to activate DREADDs. Our results support the appropriateness of these three DREADD ligands for investigating the neural mechanisms underlying motivated behavior using DREADDs. However, we found a strong sedative effect of J60 at a high dose suggesting that doses of less than 10 mg/kg should be used for behavioral tasks in which this ligand is administered i.p.

### Supplementary information

ESM 1 (PDF 937 KB)

ESM 2 (XLSX 12.1 KB)
