# Supplementary material for: Clozapine N-oxide, compound 21, and JHU37160 do not influence effortful reward-seeking behavior in mice
Source: Psychopharmacology (Berl). 2023 Oct 4;241(1):89–96. doi: 10.1007/s00213-023-06465-w (PMC10774210; doi:10.1007/s00213-023-06465-w)
Supplement: Supplementary file 1 — ESM 1 (PDF 937 KB) [file 213_2023_6465_MOESM1_ESM.pdf]

Supplementary Figures for

Clozapine N-oxide, Compound 21, and JHU37160 do not influence effortful reward-seeking behavior in mice

Yoshiatsu Aomine[1,2†], Yoshinobu Oyama[1,2†], Koki Sakurai[2,3], Tom Macpherson[1,2],

Takaaki Ozawa[1,2*], Takatoshi Hikida[1,2*]

[1]Laboratory for Advanced Brain Functions, Institute for Protein Research, Osaka University, Suita, Osaka, Japan

[2]Department of Biological Sciences, Graduate School of Science, Osaka University, Toyonaka, Osaka, Japan

[3]Laboratory of Protein Profiling and Functional Proteomics, Institute for Protein Research, Osaka University, Suita, Osaka, Japan

[†]These authors contributed equally

***Correspondence:**

Takaaki Ozawa

takaaki.ozawa@protein.osaka-u.ac.jp

Takatoshi Hikida

hikida@protein.osaka-u.ac.jp

Other Supplementary Material for this manuscript includes the following:

“Supplementary_Table1.xlsx” Results of post hoc tests of all experiments

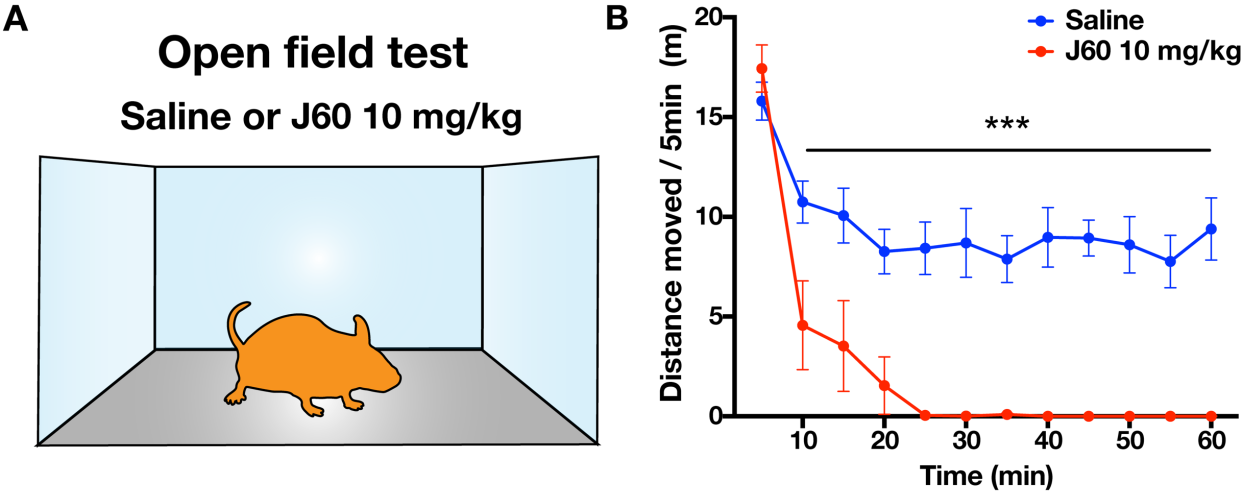

Supplementary Figure 1: J60 at a high dose (10mg/kg) completely inhibits spontaneous locomotion. (A) Illustration of the open field test. (B) Distance moved in the 60 minutes following drug administration. The graph shows the distance moved per 5-minute bin. Data represent the mean ± SEM, ***$p \leqq 0.0001$, Holm-Šídák's multiple comparisons tests.

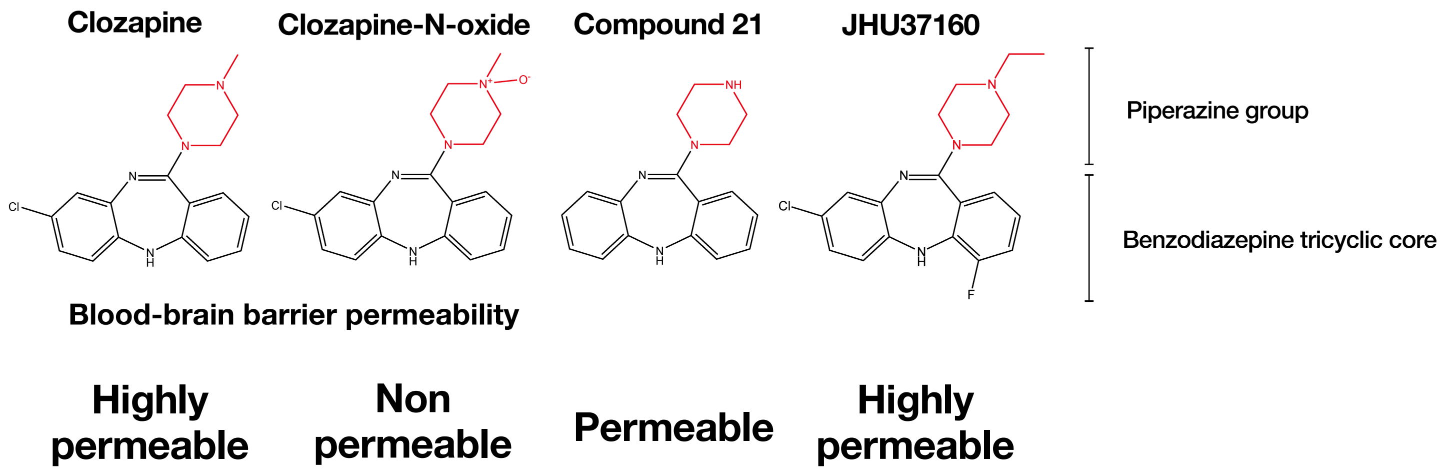

Supplementary Figure 2. The chemical structure of each DREADD ligand and blood-brain barrier permeability.
